# Toward a science-based classification of processed foods to support meaningful research and effective health policies

**DOI:** 10.3389/fnut.2024.1389601

**Published:** 2024-07-03

**Authors:** Paula R. Trumbo, Rachel Bleiweiss-Sande, Jessica K. Campbell, Eric Decker, Adam Drewnowski, John W. Erdman, Mario G. Ferruzzi, Ciaran G. Forde, Michael J. Gibney, Julie M. Hess, David M. Klurfeld, Marie E. Latulippe, Lauren E. O’Connor, Kristin J. Reimers, Barbara J. Rolls, Jackie Schulz, Connie Weaver, Lynn Yu

**Affiliations:** ^1^Paula R. Trumbo Consulting, Mount Pleasant, SC, United States; ^2^School of Health Sciences, Liberty University, Lynchburg, VA, United States; ^3^Bell Institute of Health and Nutrition, General Mills, Golden Valley, MN, United States; ^4^Department of Food Science, University of Massachusetts, Amherst, MA, United States; ^5^Department of Epidemiology, University of Washington, Seattle, WA, United States; ^6^Department of Food Science and Human Nutrition, University of Illinois at Urbana-Champaign, Urbana-Champaign, IL, United States; ^7^Arkansas Children’s Nutrition Center, Department of Pediatrics, University of Arkansas for Medical Sciences, Little Rock, AR, United States; ^8^Division of Human Nutrition and Health, Wageningen University, Wageningen, Netherlands; ^9^Institute of Food and Health, University College Dublin, Dublin, Ireland; ^10^USDA-ARS Grand Forks Human Nutrition Research Center, Grand Forks, ND, United States; ^11^Department of Applied Health Science, Indiana University School of Public Health-Bloomington, Bloomington, IN, United States; ^12^Institute for the Advancement of Food and Nutrition Sciences, Washington, DC, United States; ^13^Beltsville Human Nutrition Research Center, Agricultural Research Service, United States Department of Agriculture, Beltsville, MD, United States; ^14^Conagra Brands, Chicago, IL, United States; ^15^Department of Nutritional Sciences, The Pennsylvania State University, University Park, PA, United States; ^16^Griffith Foods, Inc., Alsip, IL, United States; ^17^College of Health and Human Services, School of Exercise and Nutritional Sciences, San Diego State University, San Diego, CA, United States; ^18^The Kraft Heinz Company, Chicago, IL, United States

**Keywords:** formulation, nutrient adequacy, safety, ultraprocessed foods, consumer acceptability

## Abstract

Processed foods have been part of the American diet for decades, with key roles in providing a safe, available, affordable, and nutritious food supply. The USDA Food Guides beginning in 1916 and the US Dietary Guidelines for Americans (DGA) since 1980 have included various types of commonly consumed processed foods (e.g., heated, fermented, dried) as part of their recommendations. However, there are multiple classification systems based on “level” of food processing, and additional evidence is needed to establish the specific properties of foods classified as “highly” or “ultra”-processed (HPF/UPFs). Importantly, many foods are captured under HPF/UPF definitions, ranging from ready-to-eat fortified whole grain breakfast cereals to sugar-sweetened beverages and baked goods. The consequences of implementing dietary guidance to limit all intake of foods currently classified as HPF/UPF may require additional scrutiny to evaluate the impact on consumers’ ability to meet daily nutrient recommendations and to access affordable food, and ultimately, on health outcomes. Based on a meeting held by the Institute for the Advancement of Food and Nutrition Sciences in May 2023, this paper provides perspectives on the broad array of foods classified as HPF/UPFs based on processing and formulation, including contributions to nutrient intake and dietary patterns, food acceptability, and cost. Characteristics of foods classified as UPF/HPFs are considered, including the roles and safety approval of food additives and the effect of food processing on the food matrix. Finally, this paper identifies information gaps and research needs to better understand how the processing of food affects nutrition and health outcomes.

## 1 Introduction

Processing of foods has existed since prehistoric times when foods were fermented, preserved with salt, sun dried, and cooked using various types of what are now considered traditional methods. Among the chief methods of food preservation to prevent bacterial and other microorganism growth were the addition of salt and sugar.

There are various definitions of food processing. In the US, one definition of processed food is “… any food other than a raw agricultural commodity and includes raw agricultural commodity that has been subjected to processing, such as canning, cooking, freezing, or milling” (1). The Institute of Food Technologists also recognizes methods such as storing, filtering, fermenting, extracting, concentrating, microwaving, and packaging, as processing methods (2). There are various food processing and packaging methods that are used both by the food industry and in home food preparation (Table 1) (3). These processes improve taste, improve or preserve nutritional content, preserve product integrity and quality, and confer other food attributes (both potentially positive and negative) by transformation through processing and formulation. The “degree” of processing can vary greatly, with degree referring to the extent to which the end-result has been modified from an agricultural product. The terms “highly processed” or “ultra-processed” are not always correlated with the amount of processing or number of processing steps to which a food is subjected. For example, flours and dairy products undergo numerous processing operations (steps) but are often classified as minimally processed (6). The formulation of foods (the recipe) is distinct from processing operations and can include incorporation of ingredients such as fat, carbohydrates, proteins, spices, herbs, flavors, vitamins and minerals for fortification and substances approved for use in food products that enhance safety and decrease spoilage.

**Table 1:** Methods used to process and package foods at home and/or during industrial production (3–5).

Processing Methods Used Both At Home and During Industrial Processing	Processing Methods Used Primarily in Industrial Processing
Heating	Irradiation
Peeling	Extrusion
	Milling
Cooling	Ultra-high temperature pasteurization
Freezing	
Fermenting	
Drying	
Smoking	
**Materials used both at home and for industrial packaging**	**Materials used only for industrial packaging**
Metal Glass Plastic Paper	Pouch Recycled materials Bioplastics

Within the past 15 years, various food classification systems have been developed to categorize foods based on what is described as the degree of processing, but the categorizations typically refer less to steps of processing and more to formulation (ingredients or added nutrients or additives) (Table 2) (7–12). Foods that are considered “highly processed” (HPF) have various characteristics depending upon the defining authority. Health Canada considers HPF to be “processed or prepared foods and drinks that add excess sodium, sugars, or saturated fat to the diet,” thus focusing on nutrient composition and food formulation rather than characteristics related to processing or processing steps (7).

**Table 2:** Food classification systems used to categorize processed food.

System	Category term	Number of categories	Description
Health Canada (7)	Highly processed	Not Applicable^a^	Add excess sodium, sugars or saturated fats to the diet
Nova (8)	Ultra-processed	Four	Formulations of industrial ingredients and substances derived from foods
UNC (9)	Highly processed	Four	Multi-ingredient industrially formulated mixtures; no longer recognizable
IARC (10)	Highly processed	Four	Industrially prepared, needing little domestic preparation
IFPRI (11)	Highly processed	Three	Secondary processing into readily edible forms, likely with high added sugars, fats, or salt
IFIC (12)	Ready-to eat processed	Five	Packaged and store-prepared, with high added and total sugars and low fiber

a Health Canada does not provide categories beyond the description that is provided here.

Several different food classification systems including Nova (8), the University of North Carolina (9), and the International Agency for Research on Cancer (10) do not specifically mention nutrient composition, but characteristics include industrial-level processing. This description of processing is not detailed further, for example, with type or number of processing steps or if the processing method differs from one used in home cooking (Table 2). The Nova classification system first introduced the term “ultra-processed” to capture both industrial scale food processing and certain aspects of food formulation, and others, such as the Siga system, have adopted and adapted the Nova approach (13). While there are multiple classification systems for HPF/ultra processed foods (UPFs), here, we focus largely on the Nova system since it has been commonly utilized for research purposes and policy development in some countries. In addition, although the Nova system incorporates considerations for the broad societal impacts of the food system, this paper’s perspective is a focus on the health impacts of the foods as consumed, in alignment with observational and intervention studies that associate consumption of foods with health outcomes.

Since 2014, some national dietary guidelines have included the Nova food classification system as a framework for dietary recommendations, including several Latin American countries, Israel, and Malaysia, among others (14–20) (Table 3). Other national guidelines, such as those from Canada, recommend limiting HPFs (21). In contrast, the United Kingdom Scientific Advisory Committee on Nutrition (SACN) recently concluded that food classification systems based on processing level are inconsistent or lack clarity on the various food components of concern (22). In particular, the Nova categories (defined below) were considered broad, leading to discordance with other nutrient or food-based recommendations. Furthermore, the SACN noted that it is unclear to what extent observed associations between UPFs and adverse health outcomes are explained by established relationships between nutritional factors and health outcomes because nutrient content was not considered, and potential confounding factors may not be accounted for (22). More recently, the 2023 Nordic Council of Ministers reviewed evidence linking foods classified as UPFs with adverse health outcomes and concluded that recommendations specific to the UPFs concept would overlap or conflict with other guidance on various types of processed foods. For example, foods high in added sugars, such as sugar-sweetened beverages and baked goods, “should be limited” and whole grain cereals should be preferentially used (23).

**Table 3:** National-level dietary recommendations and statements related to processed or ultra-processed foods.

Organization	Statement or recommendation related to ultra-processed foods^a^
Health Canada (21)	“Limit highly processed foods. If you choose these foods, eat them less often and in small amounts.”^b^
UK Scientific Advisory Council on Nutrition (SACN) (22)	“Consumption of (ultra-) processed foods may be an indicator of other unhealthy dietary patterns and lifestyle behaviours. Diets high in (ultra-) processed foods are often energy dense, high in saturated fat, salt or free sugars, high in processed meat, and/or low in fruit and vegetables and fibre. It is unclear to what extent observed associations between (ultra-) processed foods and adverse health outcomes are explained by established nutritional relationships between nutritional factors and health outcomes on which SACN has undertaken robust risk assessments.”
Nordic Council of Ministries (23)	“Despite the observed association between ultra-processed foods as a category and health outcomes, the NNR2023 Committee decided not to formulate any specific recommendations on ultra-processed foods.”
US Dietary Guidelines for Americans (24)	“Common characteristics of dietary patterns associated with positive health outcomes include … relatively lower consumption of red and processed meats, sugar-sweetened foods and beverages, and refined grains.”
Brazil (14)	“Avoid ultra-processed foods.” “Ultra-processed foods have an unbalanced nutritional composition.” “Ultra-processed foods promote excessive consumption of dietary energy.”
Uruguay (15)	“Base your diet on natural foods, and avoid the regular consumption of ultra-processed products with excessive contents of fat, sugar and salt.”
Chile (20)	“Avoid ultra-processed products with “HIGH IN” labelling.”
Peru (17)	“Protect your health avoiding the consumption of ultra-processed foods.”
Israel (18)	“The diet must be varied and based mainly upon … unprocessed food or food that has undergone minimal processing.” “It is recommended to … prefer preparing food at home from raw materials rather than ready-made food or ultra-processed food.” “It is recommended to reduce consumption of the following foods as much as possible … ultra-processed foods containing large amounts of additives such as salt/sugar or their non-natural substitutes.”
Malaysia (19)	“Limit intake of processed and ultra-processed foods.”

a In cases where “ultra-processed foods” are not mentioned in the guidance, statements on processed foods are noted.

b “Highly processed foods” are defined as “processed or prepared foods and drinks that add excess sodium, sugars, or saturated fat to the diets of Canadians”.

The 2025–2030 US Dietary Guidelines Advisory Committee (DGAC) is the first one that will specifically conduct a systematic literature review on the question: “What is the relationship between consumption of dietary patterns with varying amounts of ultra-processed foods and growth, body composition, and risk of obesity?” (24). However, the categorization of foods as UPFs will not be considered as part of the Food Pattern Modeling approach because operationalizing the “UPF” categorization is challenged by varying definitions and limitations of their application to the USDA food composition databases (25). The DGA have yet to recommend guidelines based on any level of food processing, aside from the recommendation to choose less processed forms of meats and poultry. However, in the case of processed meats, guidance is based on nutritional content (e.g., fat) or the presence of some food additives (e.g., nitrites and salt). As the DGAC follows a systematic evidence-review process, the approach to evaluation of studies that vary in the ways that foods are classified according to Nova will provide data driven insights.

Despite growing interest in limiting the consumption of foods classified as HPF/UPFs, there remain significant knowledge gaps, understanding the mechanisms by which this broad category of foods may play a causal role in health, beyond the known associations between nutrients to limit and diet-related non-communicable diseases. This paper, in part, includes information from a cross-stakeholder meeting organized by the Institute for the Advancement of Food and Nutrition Sciences (IAFNS) held in May 2023, “Considerations for Formulation and Degree of Processing in Food Classification Systems that Support Research.” It presents perspectives on the use of classification systems that may or may not incorporate processing and formulation, and various outstanding questions related to their use in constructing diets that support health. Specifically, perspectives are provided on the contribution of foods classified as HPF/UPFs to the nutrient intake and dietary patterns of Americans. Also discussed are the available studies on HPF/UPFs and disease risk and body weight, the roles and safety approval of food additives in the American diet, the effect of food processing on the food matrix, and consumer use of processed foods in the American diet. Finally, information gaps and research needs are identified to better understand how food processing and formulation affect nutrition and health outcomes, to ensure that policies based on food classification systems that incorporate processing are best positioned for positive impact.

## 2 Highly processed and ultra-processed food classification systems

Various schemes to identify foods classified as HPF/UPFs have been developed with the intent to improve the nutritional quality and healthfulness of diets (26) (Table 2). The terminology and description of each category within these classification systems varies. As described in more detail by de Araujo et al. (26), the foods represented in the individual food categories of these systems also vary. For example, the percent contribution of certain foods categories as HPFs/UPFs by a Portuguese adult population using different food classification systems ranges from 10 to 47% (26).

The food classification system based on processing that has received the most attention is Nova (27). Nova is the only internationally recognized classification system that uses the “ultra” terminology to capture both processing and formulation. With the Nova system, there are four categories of foods based on degree of processing (8). These categories capture foods, food products, culinary ingredients, and spices. Formulation of foods, i.e., combining raw materials (e.g., eggs, milk, and flour), ingredients (sugars, fats, oils), and food additives, is a major component of the Nova description of UPFs. As such, a food that is considered minimally processed according to other definitions may be classified as a UPF if it contains a food additive. However, the intention and function of the food additive is taken into consideration when classifying foods according to Nova. Additives included for the function of preservation can place foods into Group 3 and additives provided for the function of “cosmetic reasons” place foods into Group 4 (UPF). Foods that contain additives for enrichment purposes only are placed into Group 1 (11, 27). One can appreciate the challenges of determining additive function because this type of information is often not provided on food labels, and a single additive can have multiple roles (e.g., antioxidants can protect flavor, preserve color, and inhibit the formation of potentially toxic compounds). Further, differences in function of an additive would likely not translate to a differential biological response relevant to disease risk. Foods classified as UPFs are heterogeneous and undergo varying processing treatments, have different formulations, and a wide range of nutrition composition. Currently, there is a lack of a universally accepted definition for either HPFs or UPFs. Recently, more detailed guidance on how best to classify foods according to Nova has been published (28), however the information needed for accurate classification is often not available (e.g., the function of the food additive), thus requiring subjective application in research or diet evaluation. These nuances have implications for translation to policy and consumer messaging.

To improve the nutritional profile (or improve nutrient density) of foods and beverages, food manufacturers have innovated, reformulated, and introduced new foods to reduce, for example, the content of sodium, sugar, or saturated fat, as well as increase nutrients and food groups to encourage, including fruits, vegetables, vitamins, minerals, dietary fiber, and whole grains. This reformulation has required application of many technologies and processes. By nature of the Nova UPFs definition, reformulation of a food classified as UPFs will not necessarily result in a non-UPF product (e.g., lowering sugar content by the removal of sugars or replacement of sugars with non-caloric sweeteners in a sugars-sweetened beverage or the reduction of saturated fat from dairy foods) (29). Therefore, a food can remain classified as a UPF even after reformulation to reduce energy, saturated fats, added sugars or sodium (30). In parallel, the reformulation efforts and required processing toward positive nutrients and food groups for any given product could result in its categorization as a UPF. For example, yogurt that is sweetened with a low-calorie sweetener is considered UPF even though the caloric content is similar to unsweetened yogurt. However, nutrient content is not inherently related to industrial processing (31), as in fact with Nova, sodium, saturated fat, and sugars are not limited in home-prepared foods.

## 3 Contribution of foods classified as highly processed and ultra-processed to nutrient intake and dietary patterns aligned with the dietary guidelines for Americans

The Nova UPF category captures some foods that are nutrient-dense using validated scoring systems. Using two different nutrient-density scoring systems (i.e., the Health Star Rating and Nutri-Score), 21% of foods classified as UPFs received a high/healthy rating; whereas only 6% were classified as being unhealthy, with other foods falling elsewhere along the continuum (32). Minimally processed (Nova 1) foods generally scored healthier than UPFs (Nova 4). Processed foods (Nova 3), scored healthier than culinary ingredients (Nova 2), however this comparison is somewhat limited as culinary ingredients are rarely consumed on their own. Food Compass Score (FCS) is a nutrient profiling system that scores foods between 1 to 100 (being most healthful). Using FCS, 47% of Nova 4 foods had FCS < 30, 40% had FCS of 31–69, and 12.8% has FCS > 70 (32). These examples show that the Nova categories do not necessarily correlate with the nutritional quality of a product when measured using existing validated scoring systems for nutrient density. It should be noted that FCS has been the subject of some criticism (33).

Several researchers have applied the Nova system to the National Health and Nutrition Examination Survey (NHANES) and its food survey companion database, What We Eat in America (WWEIA). Based on a 2001–2018 NHANES analysis, the percent of foods classified as UPFs consumed by Americans ranged between 40 and 60 percent, depending on the age category (34). This is not surprising as it has been estimated that over 70% of the US food supply is ultra-processed when using the Nova definition (35), whereas only 4% of the food supply is not processed or is minimally processed (32). The 2020–2025 DGA notes that the average Healthy Eating Index score for Americans ages 2 years and older is 59 of a possible 100 (36). Acknowledging that categorization of foods in WWEIA according to Nova is complicated by the lack of needed detail, Martinez Steele et al. (28) recently released guidance to standardize approaches, the application of which may reduce subjectivity in future studies.

The 2020–2025 DGA includes the USDA Food Patterns (e.g., Healthy U.S.-Style Eating Pattern), which provide the recommended daily amount for each food group (e.g., vegetables, fruits) and food subgroup (e.g., dark-green, red, and orange vegetables, legumes, starchy vegetable, other vegetables) (36). These food groups and subgroups are determined by considering the types and proportions of foods Americans typically consume but in nutrient-dense forms and appropriate amounts (36). The USDA Food Patterns are based on meeting the Dietary Reference Intakes (DRIs) for individual nutrients. Many of these DRIs are based on meeting the daily individual requirement of an essential nutrient (e.g., vitamin C and iron) while others are based on reduction of chronic disease risk (e.g., dietary fiber and cardiovascular disease, sodium, and high blood pressure).

The USDA Food Patterns provide a model of how individual foods can be consumed on a daily or weekly basis and within calorie limits to achieve the DRIs for the various nutrients. The Nova UPF food category is broad and encompasses a variety of foods and food groups with diverse nutrient compositions. Although no evidence for this is currently available, removing or limiting this broad food category from the American diet could also affect the intake of nutrients that are lacking or limited in consumer’s diets, as noted below. Researchers at USDA-ARS Grand Forks Human Nutrition Research Center evaluated the feasibility of developing a menu that aligns with a healthy dietary pattern (i.e., Healthy U.S.-Style Eating Pattern) from the 2020–2025 Dietary Guidelines for Americans aiming to include 80 percent or more of calories from UPFs as defined by Nova. The investigators were able to design a diet with 91 percent of calories sourced from foods classified as UPF, as part of a 2,000 calorie diet for seven days, that met the 2020–2025 DGA recommendations and received a high Healthy Eating Index score (i.e., 86 out of 100) (37). However, the score for sodium was poor at zero, and reflected higher intakes of sodium than the average American currently consumes. It is possible that using low-sodium options for some of the UPFs modeled could improve the sodium component score. This also highlights opportunities for reductions in the DGA-designated nutrients to limit in processed foods available to consumers in the US. This study suggests that more work is needed to ensure that Nova or other systems based on “processing” are aligned with other validated metrics for a healthy diet, particularly considering the modern food supply.

Foods classified as Nova UPFs include foods that contain added ingredients that serve various roles in the finished product (i.e., deliver color, flavor, provide stability, etc.) (8). The nutritional value of a food is often associated with the use of food additives during the formulation and processing of the product as these ingredients can directly deliver nutrients (enrichment and fortification), preserve the stability of nutrients and bioactives through processing and shelf-life, create gluten-free products, replace allergens, and meet nutrient requirements for infants. Ingredients, including nutrients, may be added to foods for various technical or public health benefits at levels that are safe for the food’s intended use (38). Important nutrients that are added to foods as food additives for nutritional and public health benefits include iodine to salt (39), folic acid to certain foods classified as UPF including breakfast cereals (40) and vitamin D to fruit juices (41), soy products (41, 42), and milk (41). Vitamin D is naturally abundant primarily in fatty fish. Therefore, vitamin D fortification expands the number of foods, some of which are classified as UPF (e.g., yogurt with sweeteners, breakfast cereals) that are contributors to intake of this nutrient and enhances the opportunity for wider accessibility for this shortfall nutrient among Americans (43). Furthermore, various added dietary fibers (e.g., soluble corn fiber, inulin), which have beneficial physiological effects, contribute to the total dietary fiber intake from foods (44). While it is voluntary to enrich food products, many flours that are in food classified as UPFs are enriched and therefore must meet the standards of identity for an enriched product to contain certain levels of thiamin, riboflavin, niacin, iron, and folic acid (45). Ready-to-eat cereals are an example of a food classified as a UPF that is usually fortified with a variety of vitamins and minerals and has an impact on the nutrient intake of Americans. Approximately 19% of US adults consume ready-to-eat cereals and were reported to have a similar level of energy intake as non-eaters, but significantly higher intake of dietary fiber, and several vitamins and minerals, such as calcium, iron, magnesium, potassium, zinc, vitamin A, thiamin, riboflavin, niacin, vitamin B_6_, folate, vitamin B_12_, and vitamin D (46).

## 4 Foods classified as ultra-processed and chronic disease risk

There have been several meta-analyses conducted on observational studies that have evaluated the association between UPF consumption and risk of different chronic diseases, health-related conditions, and mortality. These meta-analyses reported a positive association between UPF intake and risk of obesity (47, 48), type 2 diabetes (48–50), hypertension (51), metabolic syndrome (47), cardiovascular events (52), and all-cause mortality (48, 52, 53). When the risk of type 2 diabetes was evaluated for individual UPF subgroups, the findings were mixed (30). Sauces, spreads, condiments, sugar and artificially sweetened beverages, and ready-to-eat dishes were associated with an increased risk of type 2 diabetes; whereas, cereals, packaged sweet snacks and desserts, savory snacks, yogurt and dairy-based desserts were associated with a lower risk of type 2 diabetes. It is important to note that the majority of available observational studies to date were either of cross-sectional or prospective cohort design, were not designed to answer the same question and adjusted for different covariables complicating the validity of combining them in meta-analyses, even if there was moderate or less heterogeneity. In addition, the Nova definition has evolved over time to include increasing detail and nuance as the classification system underwent review and was questioned by the scientific community. Details related to the types of preservatives, additives, or ingredients with included examples that denote a food as processed versus ultra-processed were added over time. Though these details improve the definition, they potentially change how the research community applies Nova to dietary data. For example, the original definition in 2009 uses the term “cosmetic additives” but does not go into great detail of what that category includes. More recent definitions describe additives such as emulsifiers and processing aids, among others, and researchers may not have considered “cosmetic additives” in older research because these additives also serve functional purposes (54).

Categorization in these studies often requires subjective judgement due to lack of needed information for accurate Nova classification as to which foods would fall into the UPF category, creating significant variation in UPF intake estimations. As previously mentioned, more standardized approaches have been proposed to harmonize application of the Nova scheme going forward (8).

Some foods that fall into the HPF/UPF category tend to be energy dense and relatively high in saturated fat, sugar, and sodium. Some of the observational studies on HPF/UPF and adult mortality, obesity, and health outcomes did not adjust for covariables such as energy intake, energy density, or for nutrients to limit, such as sodium and saturated fat that are also present in unprocessed foods. However, the majority of the associations between UPF intake and these outcomes remained significant and unchanged after making the necessary adjustments (55). None of the prospective cohorts used in a meta-analysis on UPF intake and risk of CVD mortality adjusted for saturated fat intake nor were the necessary adjustments made (53). As another example, of the nine studies included in the meta-analysis on UPF consumption and hypertension risk, only one study adjusted for sodium intake (urinary sodium) (51). While it has been reported that the association between UPF intake and health-related outcomes is mediated by diet quality and the effects are lost when diet is appropriately controlled for (56), a review of prospective cohorts noted that the adverse consequences of UPF consumption are independent of dietary quality or patterns (55). Therefore, it is difficult to determine whether there are health risks specific to UPF intake beyond recommendations to reduce sodium, saturated fat, and added sugars, and there is an opportunity for further examination of this question. Furthermore, the measured associations relied on the amount of UPF intake only and did not consider unprocessed or minimally processed food, including their nutrients and food components, as part of a total diet (54).

It is unclear whether the often-reported significant associations between HPF/UPF consumption and adverse health outcomes are the result of food processing or food composition (i.e., formulation) or factors which are already well known. For example, with increased intake of HPF/UPFs that are high in nutrients-to-limit (sodium, saturated fat, and or added sugars), a positive association would be expected between sodium intake and risk of hypertension, saturated fat intake and blood cholesterol (a marker for risk of cardiovascular disease), and added sugars and increased energy intake (with may impact weight and therefore risk of type 2 diabetes and metabolic syndrome) because the causal evidence for these relationships are well-established. However, according to Nova, certain ingredients such as butter and coconut oil are classified as “processed culinary ingredients” (Group 2). Although these ingredients include well-accepted “nutrients-to-limit” such as added sugars, saturated fat, and sodium, when used in home-prepared foods, their intake may not be accounted for in analyses that examine associations between intake of Nova 4 foods and health. This points to the opportunity for improved alignment with existing and well-established knowledge about nutrients-to-limit and health.

Consumption of foods classified as Nova 4 has been associated with lower incomes (57) and lower per calorie diet costs (58). Lower cost diets are likely to be selected by groups of lower socioeconomic status (59). Some Nova 4 foods may be more cost-effective sources of nutrients than less-processed options (60). While some studies have adjusted for income (61–63), income had a limited influence on UPF intake. Because lower-income population groups have higher rates of adverse health outcomes compared to higher-income groups (64), income is an important covariable to be included in future studies. Additional investigation could help tease out the role of HPF/UPF specifically compared to other factors that impact the health of lower-income subgroups.

Finally, because observational studies are hypothesis-generating and unable to establish causality, only well-designed clinical trials can address key outstanding questions. Additional research is needed to establish the role of food additives (nutritional and non-nutritional), individual food ingredients, the role of overall diet quality, and various types of food processes in the health outcomes that have been associated with intake of foods classified as HPF/UPF.

## 5 Sensory, hedonic, and energy density contributions to energy intake from UPFs

To date, one clinical trial has been conducted to compare energy intakes between minimally and ultra-processed diets, and related changes to body weight (65). This study was of cross-over design and conducted on 20 men and women who were offered UPFs or minimally processed foods *ad libitum* for 2 weeks each. The findings indicated a net increase in energy intake of 500 kcal per day when consuming the ultra-processed diet compared to the minimally processed diet. Although this trial was not designed to establish causal mechanisms, there has been speculation on the potential mechanisms driving observed differences in energy intake (66–68). No significant differences in metabolic markers of health were observed between the two diets, which has led to speculation that the putative mechanisms are linked to the sensory properties and eating behaviors associated with the UPF foods that influence meal size. Research has shown that meal size is most directly influenced by a food’s palatability, energy density, portion size, and the rate at which it is consumed (g/min or kcal/min) (69). The two diets in the Hall et al. (65) trial were designed to be matched for energy from fat, sugar, sodium, dietary fiber and overall diet energy density; however, when beverages were excluded, there were significant differences in energy density which was higher for the UPF diet (1.36 vs. 1.09 kcal/g, *p* < 0.0001) (70). The higher energy density of the non-beverage foods in the UPF diet likely contributed to the observed excess energy intake, and extensive research to date supports a role for energy density in promoting sustained increases in energy intake (71). While there were no significant differences in participant’s average rated palatability between the foods in the UPF and minimally processed diets, a subsequent secondary analysis reported that the UPF diet contained more items classified as “hyperpalatable” and these could have stimulated intake (72). Foods that can be consumed more quickly are known to promote greater *ad libitum* energy intake (73) and deliver lower satiety per kcal consumed (74). The UPF diet in the trial by Hall et al. (65) was consumed significantly faster (*p* < 0.001) than the minimally processed diet, and when this faster eating rate was combined with the higher energy density of the UPF foods, the energy intake rate increased by 50% compared to the minimally processed diet. Higher energy density and more rapid eating rates have been shown to associate with greater daily energy intakes (75, 76), yet there is a wide variability in the eating rate and energy density across all levels of processing (77). A follow-up-controlled *ad libitum* feeding trial tested the relative influence of degree of processing (ultra- vs. minimally processed) and meal texture-based differences in eating speed (fast vs. slow) on food intake. The study demonstrated that softer food textures that could be consumed at a faster rate were responsible for 21% increased food intake (grams) and 26% greater energy intake across both minimally and ultra-processed meals (78). Further research is now underway to formally test the role of eating rate in moderating energy intakes from ultra-processed diets (79). Findings to date suggest that energy density, food form/texture, associated eating rate, and individual differences in meal preferences are likely to be associated with the higher energy intakes reported in the clinical trial conducted by Hall et al. (65). More controlled feeding trials are needed to quantify the relative influence of these factors and identify mechanisms that can explain the observed differences in food intake between minimally versus ultra-processed diets.

## 6 Approved food additives and contaminants in highly/ultra-processed formulations: roles and safety assessment

Food additives are highly varied. Some, like cinnamon, are commonly found in home kitchens, whereas others are not. Importantly, additives are ingredients, and do not indicate a level of processing. The US Food and Drug Administration (FDA) has approved or not objected to the addition of thousands of nutrients and other compounds to foods for various functional purposes (38). These additives include table salt, sugars, fats, spices, flavorings, preservatives, starches, fatty acids, and caffeine, as well as those listed as examples in Monteiro et al. (80) as indicators of Nova category 4 foods, such as hydrolyzed proteins, soy protein isolate, gluten, casein, whey protein, various forms of added sugars, dextrose, soluble or insoluble fiber, hydrogenated or inter-esterified oil, flavor enhancers, colors, and emulsifiers, among other ingredients. Food additives are carefully evaluated by FDA for safety and regulated or are considered generally recognized as safe (also called GRAS) among qualified experts. The safety reviews require an exposure analysis of the food additive in the US population to understand current intakes and the contribution of the food additive to total intake levels. Federal regulations require evidence that each substance is safe at its intended level of use before it may be added to foods. Furthermore, all additives are subject to ongoing safety reviews as scientific understanding and methods of testing continue to improve. If new information becomes available on the safety of a food additive, the FDA re-evaluates the data for making a new determination. As one example, in 2018, FDA revoked approval of 6 synthetic flavoring substances and enhancers because of evidence for cancer in laboratory animals (81). Conversely, FDA maintained the safety of aspartame after recent re-examination (82).

It has been stated that foods classified as UPFs contain contaminants, such as polycyclic aromatic hydrocarbons and acrylamide, which are associated with adverse health effects (83, 84). These contaminants, however, are produced at any level of processing, performed at the industrial level or through home food preparation and the Nova or other systems do not provide information on the levels of contaminants for each category to understand the distinction. In fact, home-cooked prepared foods can contain higher levels of acrylamide than highly processed foods (85). Generally, acrylamide levels in foods are highly variable, depending upon factors such as agricultural practices, food preparation, and cooking method, and data are not adequate to point specifically to foods classified as UPF as the culprit (86). A significant advancement in these discussions would include identification of specific components of foods classified as HPF/UPFs that are associated with potential negative health outcomes, to include differentiation and quantification of adverse nutrients, food additives and contaminants.

In addition to ongoing safety testing of food additives, future studies should also consider evaluating effects on the microbiome, once a microbiome associated with better health is established. Food additive emulsifiers have been linked to inflammatory bowel disease (IBD) (87) and understanding the impact of such food additives on IBD could provide valuable mechanistic information. Furthermore, there is observational data demonstrating an association between the consumption of emulsifiers and increased risk of certain cancers (88).

Highly/ultra-processed foods are typically sold in packaging of which the form and composition is highly variable. Because food is in contact with materials used in packaging, assessment of human exposure to these materials through consumption of packaged foods is of importance. A recent study that focused on baby food containers and reusable food pouches showed that nanoplastics were released into foods after 6 months at room temperature or refrigeration and several-fold more after microwaving (89). Although the health implications of various levels of exposure to micro- and nanoplastics (MNP) requires additional research, microplastics have been found in human thrombi of individuals treated by thrombectomy, and the severity of disease (ischemic stroke, myocardial infarction, or deep vein thrombosis) was associated with higher MNP concentrations (90).

Food classification systems would be most productive if aligned with the existing science, including thresholds for use and contaminant levels in foods apart from the location of food production. Without this, categorization is based on a list of predetermined food ingredients that serve as qualitative markers of the Nova Group 4 category.

## 7 Is the food matrix and nutritional quality impacted by processing?

The metabolic consequences of food intake assume food composition is the sum of its parts, but do not account for underlying differences in food matrix structure and subsequent bioavailability of nutrients for digestion and absorption (91). The food matrix is the complex micro- and macrostructural environment in which the various components of a food or product interact, and research has shown that the same nutrients behave very differently depending on food matrix structure (92). In many cases, food components cannot be consumed or digested in the absence of processing and changes in the food matrix structures are necessary to promote consumer appeal, digestibility, and bio-accessibility of a food’s nutrients (93). For example, milling of cereals, gelatinization of starch during cooking of grains and denaturation, and precipitation of protein in egg whites by heat processing are all required to optimize digestion of these raw materials (91). Some have speculated that the degree of food processing affects food matrices, which in turn may exhibit a deleterious effect on human health (94). Processing can change the physicochemical properties of the food matrix, such as polysaccharide gelling, protein denaturation, water and electrolyte loss, increasing/decreasing the content of antinutrients including phytates, and degradation of vitamins and bioactive compounds (95–97). For example, extensively refined grains may have an increased glycemic index due to the removal of the bran layer and an altered food matrix (98). Two foods with identical composition can differ in functionality and have distinct metabolic and physiological impact on consumption (99). For example, an early study showed differing glycemic and insulin response after applesauce consumption compared to whole apples (100). Some modern processed foods contain purified or isolated fractions, such as protein isolates, and enzymatically modified ingredients. This has been suggested to increase the biochemical complexity and diversity of nutritional components in the modern diet (101).

Processing of plant-based foods can induce some changes in the food matrix, which may improve bio-accessibility and bioavailability of its components (e.g., polyphenols and carotenoids), by mechanical, thermal or by chemical transformation (102, 103). Briones-Labarca et al. (104) subjected apples to a high pressure of 500 megapascal pressure unit for 2, 4, 8, and 10 min and found an increase in antioxidant capacity over the digestion period, demonstrating that high pressure processing favored the release of antioxidants in the small intestine. Lycopene bioavailability has also been routinely shown to be higher from processed tomato products than from raw tomatoes (105). Within the dairy product range, processing and matrix structure may enhance interactions between nutrients and modify the metabolic effects of dairy consumption (106). For example, milk is pasteurized to remove pathogenic bacteria, and homogenized to subdivide fat globules and decrease physical separation of fat which, in turn, can alter the temporal rates of flavor, protein, and lipid release during consumption and digestion (107). Research shows that dairy fat, when consumed in the form of cheese, affected blood lipids differently than when the constituents were eaten in different matrices. Consuming fat within a cheese matrix resulted in significantly lower blood cholesterol levels compared to an equivalent fat intake as butter (108, 109). As such, the relationship between types of processing operations of raw materials and subsequent health outcomes is process and context specific. Whereas, some concerns remain regarding neo-formed compounds and potential negative side-effects of processing, there are important food safety, digestibility, palatability, and nutrient bioavailability benefits that also need to be considered (6).

## 8 Consumers’ use of highly/ultra-processed foods

Several factors influence consumer purchasing and acceptance of certain foods, including taste/palatability, cost/value, and ease of preparation/convenience (110). These factors are important to consider when making food-based recommendations to ensure recommendations are achievable. Foods have more meaning to consumers than the sum of their food components (110). Enjoyment of food needs to be considered if healthier products are to be accepted and to encourage long-term adoption of healthier, more sustainable diets. Cooking at home does not necessarily translate to preparation of healthier foods, and meals cooked from scratch can be more indulgent than pre-prepared dishes (30).

Taste is an important factor for consumer purchasing and acceptability (110). Some additives are used to make foods more palatable and appealing (e.g., flavors, flavor enhancers, emulsifiers, colors, texturants and stabilizers). For example, non-nutritive sweeteners are used instead of sugars to add a sweet taste to a food without adding calories. Stabilizers, such as guar gum, gum arabic, and locust bean gum, help to preserve the structure of the food, and are also dietary fibers that provide beneficial physiological effects (44). Whole wheat breads commonly use processing steps such as enzyme treatments and food additives such as emulsifiers, antimicrobial agents, and sugars to increase palatability and increase consumption rates (111), while also increasing shelf-life and reducing food waste.

## 9 Accessibility and affordability of foods classified as highly/ultra-processed

Highly or ultra-processed foods can cost less than minimally processed (more perishable) foods (112). In one evaluation, foods classified as UPFs were shown to cost approximately $0.55/100 kcal; whereas minimally processed foods cost $1.45/100 Kcal. This is because vegetables, fruits, meat, poultry, and fish products which are available in minimally processed forms had the highest cost per 100 kcal; whereas grains, mostly consumed as more processed, had the lowest cost. Hall and colleagues (65) reported that the cost of ingredients alone was approximately 50% higher for the minimally processed versus the UPF diet provided in their study.

Foods classified as HPF/UPF can be more affordable, in part, because manufacturers purchase the ingredients in large quantities, maximize yield and efficiencies to decrease waste, utilize food side stream products to offset processing costs, and utilize various processing and packaging technologies, as well as food additives (e.g., antioxidants) to maximize shelf-life (6). Affordability is an important factor considered by American consumers (110). The lowest (20%) income households spend approximately 35% of their income on food; whereas the highest (20%) income households only spend 8.2% (113).

Because of the high demands of daily life on time for activities, such as working, commuting, and/or attending to children and their activities, the use of foods that can be prepared in an easy and convenient manner is important. Preparing all foods from scratch is no longer realistic for many Americans. According to a USDA report, the average American spends only 37 min in food preparation and cleanup (114). Although specifics vary, the literature consistently indicates that time spent cooking has declined for Americans at least until the 1990s, and some sources indicate a continual decline since the 1920s (115). In a study of 1,710 young adults, the majority engaged in food preparation less than once weekly. Reasons included lack of time, lower perceived skill level, and other resource limitations (116).

If there was a shift towards the reduction of the broad category of foods classified as HPF/UPF in the diet, cost, availability and consumer acceptability and willingness to adapt would need to be considered in addition to consumer literacy in identification of foods classified as HPF/UPF. Some foods classified as UPF also provide nutrition at a lower cost (e.g., fortified cereals). Furthermore, it would be essential to understand potential changes in agricultural and productions systems so that that there is sufficient supply and distribution of a less processed food supply to enable equitable access to raw materials and products.

## 10 Discussion

More research is needed to better understand the potential beneficial and adverse effects of different levels (e.g., minimally versus highly processed/ultra-processed) and types (e.g., extrusion, fermentation) of food processing, and separately, of food formulation (e.g., nutrients, food additives, contaminants, and other food components) on nutrition and health. Processing compared to formulation effects on nutrition and health are distinct but may interact to impact health in ways that are not yet understood (117). In particular, there is a paucity of information on how specific processing methods affect the concentration, bioavailability, or other aspects of nutrients and their delivery that may be important to health. Processing may also impact the food matrix, for which there is emerging evidence of relevance for nutrient delivery, biological response, and potentially eating behavior. In addition to strong observational studies assessing hard clinical endpoints, this will require data from randomized controlled trials that are designed to evaluate the mechanisms of action (e.g., gut microbiome) and causal relationships between various food forms and components and chronic disease risk factors and toxicity. However, such studies must include a control to make adequate comparisons, such as foods with the same formulations cooked using home preparation techniques.

Additionally, there is little information on the embedded costs—including ingredient, time, equipment, and energy costs—associated with procuring and consuming a largely minimally processed diet compared to a highly processed diet in the US. It will be important to determine the direct and indirect costs of diets at different processing levels for a variety of contexts, including geographic location (e.g., urban versus rural), level of access to food outlets, ingredient availability, and degree of culinary knowledge and access to cooking equipment.

A stronger evidence base, consisting of both observational studies and RCTs, will allow for a more balanced and critical review of how foods subjected to various processes influence human health to inform future evidence-based dietary guidance and impactful policies. Such studies would clarify the nutrition and health impacts of specific processing steps (linked to “highly processed” and “ultra-processed”), differentiating or comparing steps used in home preparation with those used for industrial production. In addition, ingredient (formulation) effects would ideally be considered separately because processing and ingredients confer different food characteristics and would be anticipated to impact health in different ways. A 2022 survey reported that only 46% of respondents could easily explain what processed foods are and identify examples of processed foods (118). Finally, although in theory, a nutrient-dense and balanced diet could be prepared at home on a daily basis, the realities of time, cost, convenience, consumer education and acceptance, and access as well as factors core to ensuring a global and equitable food supply such as safety, food waste, and sustainability, need to be considered in research and guidance related to foods classified as HPF/UPF and dietary inclusion or exclusion of particular food categories.

## Author contributions

PT: Writing – review & editing, Writing – original draft. RB-S: Writing – review & editing. JC: Writing – review & editing. ED: Writing – review & editing. AD: Writing – review & editing. JE: Writing – review & editing. MF: Writing – review & editing. CF: Writing – review & editing. MG: Writing – original draft. JH: Writing – review & editing. DK: Writing – review & editing. ML: Writing – review & editing. LO’C: Writing – review & editing. KR: Writing – review & editing. BR: Writing – review & editing. JS: Writing – review & editing. CW: Writing – review & editing. LY: Writing – review & editing.
